# Effects and interferences of emicizumab, a humanized bispecific antibody mimicking activated factor VIII cofactor function, on lupus anticoagulant assays

**DOI:** 10.1111/ijlh.13114

**Published:** 2019-10-31

**Authors:** Joanne I. Adamkewicz, Anna Kiialainen, Ido Paz‐Priel

**Affiliations:** ^1^ Genentech, Inc. South San Francisco CA USA; ^2^ F. Hoffmann‐La Roche Ltd Basel Switzerland

Dear Editors,

In a recent study published in *Thrombosis and Haemostasis*, we investigated the effects of the novel bispecific antibody emicizumab on a variety of in vitro laboratory coagulation tests.^1^ Emicizumab provides effective bleed prevention in persons with hemophilia A (PwHA),^2^ but due to its mechanism of action, we reasoned that it may affect tests commonly used to monitor coagulation. The goal of our study was to inform the selection and interpretation of coagulation assays for PwHA receiving emicizumab prophylaxis.^1^ We showed that emicizumab has a strong interference effect on assays based on the activated partial thromboplastin time (aPTT), such as one‐stage assays for FVIII, protein C, and protein S activity; a weak effect on the prothrombin time and derived fibrinogen assays; and no effect on various chromogenic and immunologic assays.^1^ However, there are no data currently available on the effect of emicizumab on functional assays to detect lupus anticoagulants (LAs). LAs are immunoglobulins that inhibit phospholipid‐dependent coagulation tests by binding to phospholipid cofactor proteins. While rare, the presence of LAs in PwHA is significant in that they can interfere with the ability to detect and monitor FVIII inhibitors by Bethesda assays based on aPTT, and in a few instances even with chromogenic Bethesda assays (which are recommended for management of emicizumab patients).^3^ Therefore, it is valuable to explore the effect of emicizumab on several functional assays for LA detection based on different assay principles. The intent of this letter is to supplement the findings of our previous study^1^ by investigating the effect of emicizumab on the results of LA detection assays.

To this end, we used patient plasma samples to assess the effect of emicizumab therapy on three different LA detection assays. Frozen plasma samples were sourced as follows: two from persons with severe hemophilia A without FVIII inhibitors (Precision BioLogic Inc), two samples positive for LAs (George King Bio‐Medical Inc and Precision BioLogic Inc), and CRYOcheck™ pooled normal plasma (Precision Biologic Inc). In addition, individual samples from 12 healthy plasma donors were generated (menal GmbH) via blood collection into plastic tubes containing 0.109 mol/L sodium citrate as an anticoagulant, centrifuged (2000 × *g*, 15 minutes at room temperature) and the resulting plasma was transferred to another plastic tube for a second spin. Plasma was then aliquoted, immediately frozen (−70°C), and thawed in a water bath at 37°C immediately before testing. Analyses were performed in duplicate using the commercially available diagnostic kits according to manufacturer instructions; results presented are means of the two determinations. In each experiment, emicizumab was spiked into the samples to produce final plasma concentrations of 0, 50, 100, and 150 µg/mL.

Three LA detection assays were evaluated: the aPTT‐based STA‐Staclot LA assay (Stago); the Dilute Russel Viper Venom Time (DRVVT; STA‐Staclot DRVV Screen and Confirm LA assays, Stago); and the Taipan venom time (TVT; Diagnostic Reagents Ltd).

The aPTT assay is based on hexagonal phase phospholipid neutralization of LAs.^4^ An LA‐sensitive aPTT is performed with and without the addition of hexagonal phase phospholipids, and the difference in clotting time is calculated. LAs interfere with thrombin generation in the aPTT assay by disrupting the formation of two phospholipid‐dependent coagulation factor complexes (tenase and prothrombinase). Shortening of the detected clotting time by ≥8 seconds (as per manufacturer recommendations) with the addition of hexagonal phase phospholipids was indicative of the presence of LAs. However, the assay cannot be used with plasma samples containing anti‐factor antibodies (inhibitors), as these will prolong the clotting time regardless of the presence of LAs or addition of hexagonal phase phospholipids.

The DRVVT assay is based on the activation of FX and FV using the snake venom of *Daboia russelii* (Russell's viper) with the addition of a low (DRVV Screen) versus high (DRVV Confirm) concentration of phospholipids. The assay detects LAs by their ability to interfere with thrombin activation by the prothrombinase complex. By activating coagulation downstream of factors VIII and IX, the test is not subject to being affected by their deficiencies or specific inhibitors. Per the test manufacturer's recommendation, a DRVVT screen ratio of >1.2 (test sample ÷ normal plasma pool) was indicative of the presence of LAs.

The TVT assay is based on the activation of prothrombin to thrombin by the venom of *Oxyuranus scutellatus* (Taipan snake), which is dependent on the presence of phospholipids and free calcium ions^5^; LAs prolong the TVT. TVT was performed by mixing 100 µL of sample with 100 µL of Bell and Alton phospholipid (Diagnostic Reagents, reconstituted with 5 mL of water, diluted 1:6 with imidazole buffer) and finally adding 200 µL of Taipan venom reagent (Diagnostic Reagents). The TVT clotting times were divided by the mean clotting time of 10 determinations of the normal plasma pool. A ratio >1.12 was indicative of the presence of LAs.^6^

The aPTT‐based and DRVVT LA assays were performed on the STA‐R Evolution analyzer (Stago), and the TVT assay was performed on the MC10 coagulometer (ABW Medizin und Technik GmbH). Investigations were performed at menal GmbH.

Our results showed that emicizumab substantially shortened clotting times of the aPTT‐based LA assay, both with and without the presence of hexagonal phase phospholipids (Figure 1). The presence of emicizumab also affected the difference between the clotting times, and thus could alter the assignment of samples as LA positive or negative.

**Figure 1:**
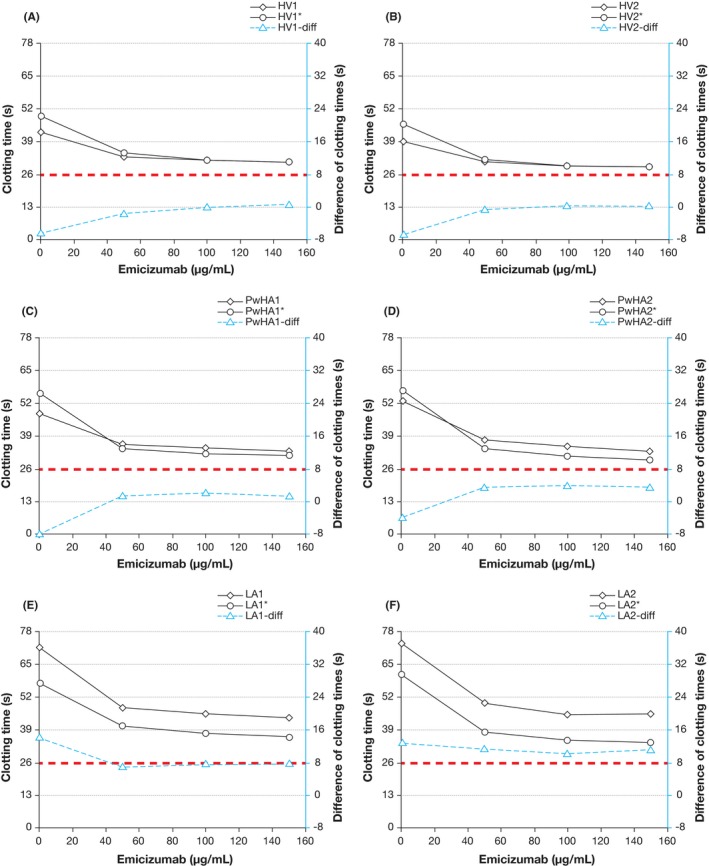
Effect of emicizumab on the aPTT‐based hexagonal (II) phase phospholipid clotting assay (Staclot LA). Two samples from healthy individuals (A and B), two samples from PwHA (C and D), and two samples positive for LAs were analyzed (E and F). Each panel (A–F) shows the dose response for emicizumab of an LA‐sensitive aPTT assay (diamonds) and the same aPTT assay with the addition of hexagonal (II) phase phospholipids, which can neutralize LAs (circles). The difference of the two aPTTs is marked with triangles (blue dashed line, secondary y axis) and is considered positive if it exceeds 8 s. Note that according to manufacturer's instructions, the test plasma is mixed with an equal volume of healthy donor plasma prior to measurement, resulting in similar baseline aPTT values for healthy donor samples (A and B) and hemophilia A samples (C and D). Footnotes: *Sample with addition of hexagonal (II) phase phospholipids. aPTT, activated partial thromboplastin time; HV, healthy volunteer (samples); LAs, lupus anticoagulant (samples); PwHA, persons with hemophilia A

Emicizumab triggered a weak but detectable concentration‐dependent prolongation of DRVVT clotting times (Figure 2A), and accordingly a slight increase in DRVVT ratios (Figure 2B). The mean increase in DRVVT with 50 µg/mL emicizumab (which corresponds to the clinical median trough plasma concentration)^2^ was 1.5 seconds (range, 1.3‐1.8 seconds) for non‐LAs samples and 7.0 seconds (range, 6.6‐7.3 seconds) for LAs samples (Table S1). The mean increase in the ratio was 0.04 (0.03‐0.05) and 0.18 (0.17‐0.18), respectively. The use of the normalized ratio (DRVVT screen ratio ÷ DRVVT confirm ratio) halved the effect of emicizumab. Emicizumab had no effect, however, on the prothrombin‐activator based TVT assay (Figure 3).

**Figure 2:**
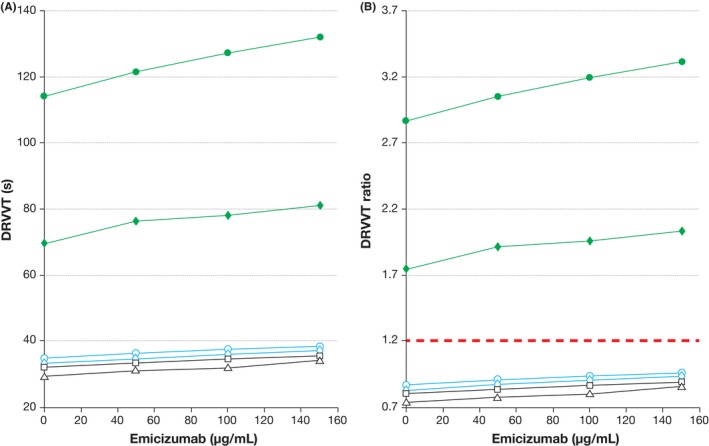
Effect of emicizumab on the DRVVT assay (STA‐Staclot DRVV Screen) expressed in seconds (A) and as ratio of the results from a normal plasma pool provided in the kit (B). Two samples from healthy individuals (black open symbols, triangle and square), two samples from persons with hemophilia A (blue open symbols, circle and diamond), and two samples positive for LAs were analyzed (green closed symbols, circle and diamond). The red dashed line in panel B (ratio > 1.2) depicts the cutoff for a positive DRVVT assay, indicative of the presence of LAs. Footnotes: DRVVT, dilute Russel viper venom time; LA, lupus anticoagulant

**Figure 3:**
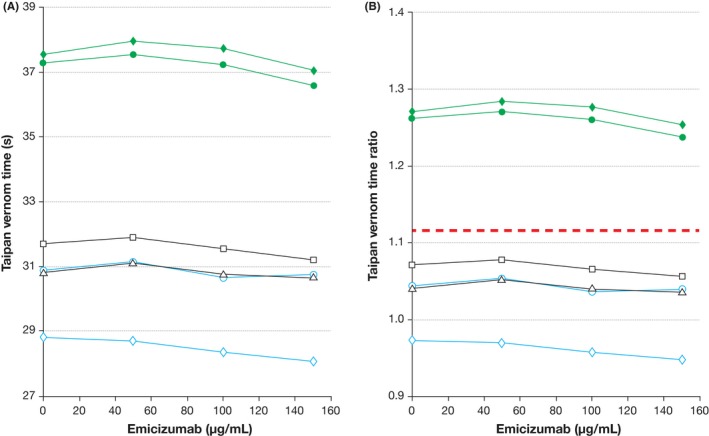
Effect of emicizumab on the Taipan venom time assay expressed in seconds (A) and as ratio of the results from the mean of 10 plasma samples from healthy volunteers (B). Two samples from healthy individuals (black open symbols, triangle and square), two samples from persons with hemophilia A (blue open symbols, circle and diamond), and two samples positive for LAs were analyzed (green closed symbols, circle and diamond). The red dashed line in panel B (ratio > 1.12) depicts the cutoff for a positive Taipan venom time assay, indicative of the presence of LA. Footnotes: LAs, lupus anticoagulant

Taken together, all three assays correctly discriminated the LA and non‐LA samples in the absence of emicizumab. However, emicizumab interfered with the Staclot LA assay to the extent that an LA‐positive sample would have been incorrectly classified as LA‐negative; this is not surprising given that emicizumab has a documented very strong shortening effect on the aPTT.^1^ Emicizumab had a weak effect on DRVVT. This effect of emicizumab on DRVVT is much smaller than the effect of direct oral anticoagulants on this assay.^7^ The mechanism of DRVVT prolongation is likely due to a weak steric interference with coagulation reactions in which FXa is generated, due to the binding of FX by emicizumab.^8^ The lack of effect of emicizumab on TVT was expected, as emicizumab acts further upstream in the coagulation cascade.

Current guidelines for the detection of LAs recommend DRVVT followed by an LA‐sensitive aPTT‐based test^9^; however, results of such work‐up are hard to interpret in the presence of emicizumab, as its interference could lead to incorrect assay results. Interference observed with aPTT‐based assays may yield false‐negative results, whereas interference with DRVVT assays may result in false positives. The TVT may be an alternative option for evaluating LA status in emicizumab‐containing samples. The assay has also been proposed as an alternative to the DRVVT for samples containing direct oral anticoagulants.^6^, ^10^ However, the assay is not widely used, nor fully standardized, and it does not have 100% sensitivity for LAs.^10^, ^11^

This exploratory study has a number of limitations: Plasma samples were derived from a small number of individual donors spiked with emicizumab, and not ex vivo samples from individuals receiving emicizumab therapy. Assay cutoffs from the literature or from manufacturers’ instructions were applied to the interpretation of the results, rather than locally generated reference ranges as recommended for clinical use.^12^ One reagent was tested for each assay; however, a number of different reagents are commercially available for DRVVT‐ and aPTT‐based LA assays, and emicizumab could have differential effects on assay outcomes using these different systems. Additionally, further assays exist for the assessment of LAs than those evaluated in our study; the dilute prothrombin time in particular may warrant investigation, as our previous work found a very small reduction in standard prothrombin time in the presence of emicizumab.^1^

In conclusion, when analyzing samples from individuals receiving emicizumab therapy, all aPTT‐based assays should be avoided, including those for LAs, although it is acknowledged that a few patients who express their LA activity exclusively via aPTT‐based LA tests could be missed. Further studies on DRVVT and TVT in the presence of emicizumab are desirable to confirm the safe and accurate use and interpretation of these assays when testing for LAs in samples containing emicizumab.

## CONFLICT OF INTEREST

JIA is an employee of Genentech, Inc, and holds stock with F. Hoffmann‐La Roche Ltd. IPP is an employee of Genentech, Inc AK is an employee of and holds stock with F. Hoffmann‐La Roche Ltd.

## AUTHOR CONTRIBUTIONS

JIA designed the study, analyzed and interpreted data, and prepared the manuscript. AK and IPP interpreted data and edited the manuscript. All authors granted final approval of the manuscript for submission.
