# Supplementary material for: Effects and interferences of emicizumab, a humanized bispecific antibody mimicking activated factor VIII cofactor function, on lupus anticoagulant assays
Source: Int J Lab Hematol. 2019 Oct 31;42(2):e71–5. doi: 10.1111/ijlh.13114 (PMC7079058; doi:10.1111/ijlh.13114)
Supplement: Supplementary file 1 [file IJLH-42-e71-s001.docx]

**Supporting information**

**Supplemental Table 1:** DRVVT screen and confirm clotting times and ratios (vs the clotting time of a normal plasma pool), as well as the normalized ratio (DRVVT screen ratio ÷ DRVVT confirm ratio) in relation to the spiked emicizumab concentration. The ratios are also expressed as a percentage of control (ie, the measurement without spiked emicizumab).

|  | **Emicizumab µg/mL** | **DRVVT screen (sec)** | **DRVVT confirm (sec)** | **DRVVT screen (ratio)** | **DRVVT confirm (ratio)** | **DRVVT normalized ratio** | **Screen ratio vs control** | **Confirm ratio vs control** | **Normalized ratio vs control** |
| --- | --- | --- | --- | --- | --- | --- | --- | --- | --- |
| **Healthy donor plasma 1** | 0 | 29.6 | 33.3 | 0.74 | 0.84 | 0.89 | 100% | 100% | 100% |
|  | 50 | 31.0 | 33.9 | 0.78 | 0.85 | 0.92 | 105% | 102% | 103% |
|  | 100 | 32.1 | 34.7 | 0.81 | 0.87 | 0.92 | 108% | 104% | 104% |
|  | 150 | 34.3 | 35.0 | 0.86 | 0.88 | 0.98 | 116% | 105% | 110% |
| **Healthy donor plasma 2** | 0 | 32.2 | 33.1 | 0.81 | 0.83 | 0.97 | 100% | 100% | 100% |
|  | 50 | 33.4 | 34.3 | 0.84 | 0.86 | 0.97 | 104% | 104% | 100% |
|  | 100 | 34.6 | 35.4 | 0.87 | 0.89 | 0.98 | 108% | 107% | 101% |
|  | 150 | 35.7 | 36.2 | 0.90 | 0.91 | 0.99 | 111% | 109% | 102% |
| **HA**  **plasma 1** | 0 | 34.6 | 33.9 | 0.87 | 0.85 | 1.02 | 100% | 100% | 100% |
|  | 50 | 36.3 | 35.1 | 0.91 | 0.88 | 1.03 | 105% | 104% | 101% |
|  | 100 | 37.4 | 36.0 | 0.94 | 0.90 | 1.04 | 108% | 106% | 102% |
|  | 150 | 38.4 | 36.8 | 0.96 | 0.92 | 1.04 | 111% | 108% | 102% |
| **HA**  **plasma 2** | 0 | 33.0 | 32.9 | 0.83 | 0.83 | 1.00 | 100% | 100% | 100% |
|  | 50 | 34.8 | 34.0 | 0.87 | 0.85 | 1.03 | 105% | 103% | 102% |
|  | 100 | 36.1 | 35.4 | 0.91 | 0.89 | 1.02 | 109% | 108% | 102% |
|  | 150 | 37.3 | 35.9 | 0.94 | 0.90 | 1.04 | 113% | 109% | 103% |
| **LA**  **plasma 1** | 0 | 114.2 | 57.2 | 2.87 | 1.44 | 2.00 | 100% | 100% | 100% |
|  | 50 | 121.5 | 59.6 | 3.05 | 1.50 | 2.04 | 106% | 104% | 102% |
|  | 100 | 127.3 | 61.5 | 3.20 | 1.55 | 2.07 | 111% | 108% | 104% |
|  | 150 | 132.1 | 63.3 | 3.32 | 1.59 | 2.09 | 116% | 111% | 104% |
| **LA**  **plasma 2** | 0 | 69.7 | 40.0 | 1.75 | 1.01 | 1.74 | 100% | 100% | 100% |
|  | 50 | 76.3 | 40.5 | 1.92 | 1.02 | 1.88 | 110% | 101% | 108% |
|  | 100 | 78.2 | 42.5 | 1.96 | 1.07 | 1.84 | 112% | 106% | 106% |
|  | 150 | 81.2 | 44.1 | 2.04 | 1.11 | 1.84 | 117% | 110% | 106% |

DRVVT, dilute Russel viper venom time; HA, hemophilia A; LA, lupus anticoagulant.
